# Supplementary material for: First Comprehensive Study of a Giant among the Insects, Titanus giganteus: Basic Facts from Its Biochemistry, Physiology, and Anatomy
Source: Insects. 2020 Feb 12;11(2):120. doi: 10.3390/insects11020120 (PMC7073837; doi:10.3390/insects11020120)
Supplement: Supplementary file 1 [file insects-11-00120-s001.pdf]

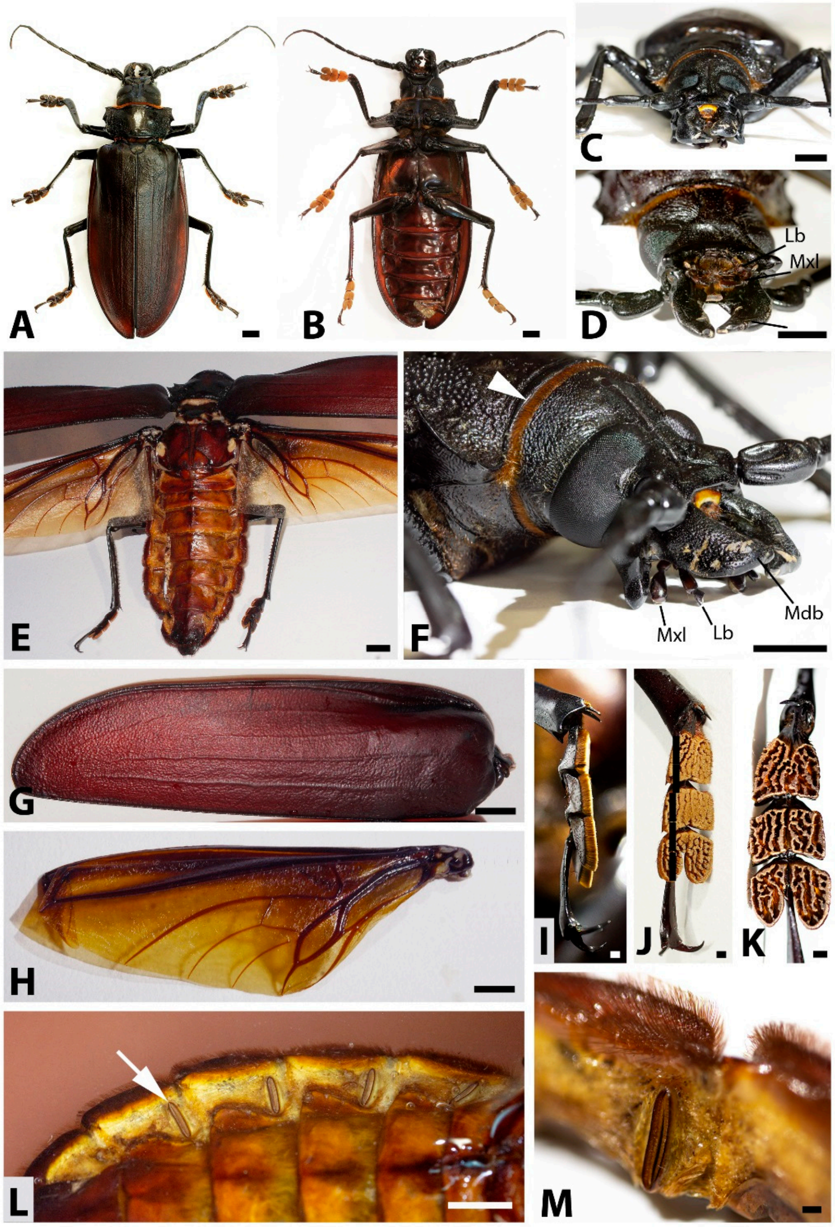

Figure S1. *T. giganteus* external morphology. (**A**,**B**)—Habitus, dorsal and ventral view. (**C**)—Anterior view. (**D**)—Head, anteroventral view. (**E**)—Posterodorsal view of body with wings open. (**F**)—Head, anterolateral view (arrow points to a row of setae lining anterior prothoracic margin). (**G**)—Left elytron, dorsal view. (**H**)—Folded left wing, dorsal view. (**I**,**J**)—Third right leg tarsus, lateral view, ventral view, respectively. (**K**)—First three tarsal segments of third right leg, ventral view on the moist adhesive setae (the moistening elicited structural artefacts). (**L**)—Left half of abdomen with spiracles (spiracle of sixth segment arrowed). (**M**)—A detailed view of sixth abdominal spiracle. Abbreviations: Mdb, mandible; Mxl, maxilla, maxillary palp; Lb, labium, labial palp. Scale bar: 1 cm (**A**–**H**,**L**); 1 mm (**I**–**K**,**M**).
